# PROTOCOL FOR LIVER TRANSPLANTATION IN UNRESECTABLE COLORECTAL METASTASIS

**DOI:** 10.1590/0102-672020210002e1625

**Published:** 2022-01-31

**Authors:** Lucas ERNANI, Eduardo de Souza Martins FERNANDES, Rodrigo Bronze de MARTINO, Fabricio Ferreira COELHO, Felipe Pedreira Tavares de MELLO, Ronaldo ANDRADE, Leandro Savattone PIMENTEL, Luciana Bertocco de Paiva HADDAD, Paulo HERMAN, Wellington ANDRAUS, Luiz Augusto Carneiro D’ALBUQUERQUE

**Affiliations:** 1 Departamento de Gastroenterologia, Hospital de Clínicas, Faculdade de Medicina, Universidade de São Paulo, São Paulo, SP, Brasil; 2 Departamento de Cirurgia Geral e Transplantes, Hospital Adventista Silvestre, Rio de Janeiro, RJ, Brasil; 3 Serviço de Cirurgia e Transplante de Órgãos Abdominais, Hospital São Lucas - Copacabana, Rio de Janeiro, RJ, Brasil.

**Keywords:** Transplantation, Liver Transplantation, Colorectal Neoplasms, Neoplasm Metastasis, Transplante, Transplante de Fígado, Neoplasias Colorretais, Metástase.

## Abstract

Colorectal cancer (CRC) is the third most common neoplasm, and half of the patients with CRC develop liver metastasis. The best prognostic factor for colorectal liver metastasis (CRLM) is the possibility of performing a resection with free margins; however, most of them remain unresectable. The justification for performing liver transplantation (LT) in patients with CRLM regards an increase in the number of resectable patients by performing total hepatectomy.

**AIM:**

The aim of this study was to provide a Brazilian protocol for LT in patients with unresectable CRLM.

**METHOD:**

The protocol was carried out by two Brazilian institutions, which perform a large volume of resections and LTs, based on the study carried out at the University of Oslo. The elaboration of the protocol was conducted in four stages.

**RESULT:**

A protocol proposal for this disease is presented, which needs to be validated for clinical use.

**CONCLUSION:**

The development of an LT protocol for unresectable CRLM aims to standardize the treatment and to enable a better evaluation of surgical results.

## RESUMO - RACIONAL

O câncer colorretal é a terceira neoplasia mais frequente e metade dos pacientes desenvolvem metástase hepática. O melhor fator prognóstico na metástase hepática de câncer colorretal (MHCCR) é a possibilidade de ressecção com margens livres, porém a maioria permanece irressecável. O racional em realizar transplante hepático (TH) em pacientes portadores de MHCCR está na ampliação do número de pacientes ressecáveis através de uma hepatectomia total.

**OBJETIVO:**

Apresentar protocolo brasileiro para realização de transplante hepático em pacientes com MHCCR irressecável.

**MÉTODO:**

O protocolo foi realizado por duas instituições com grande volume de ressecções e transplantes hepáticos no Brasil, baseado no trabalho realizado pela Universidade de Oslo. A elaboração foi dividida em 4 etapas.

**RESULTADO:**

É apresentada proposta de protocolo para esta doença a ser validada na aplicação clínica.

**CONCLUSÃO:**

Foi possível elaborar protocolo de transplante hepático para MHCCR irressecável a fim de uniformizar o tratamento e melhor avaliar os resultados cirúrgicos.

## INTRODUCTION

Colorectal cancer (CRC) is the third most common type of cancer in both genders. At the time of diagnosis, nearly 25% of patients have metastasis and the liver is the most affected organ (present in 80% of cases); it is estimated that half of the patients with CRC will develop liver metastasis at some point in the course of the disease^13^ ^,^ ^14^.

Currently, the treatment of metastatic CRC (stage IV) is based on a multidisciplinary and multimodal approach^13^ ^,^ ^18^. The possibility of performing a resection with free margins is the best prognostic factor for colorectal liver metastasis (CRLM)^14^. In this scenario, hepatectomy has become the main treatment of CRLM, having an overall survival rate of 30-55% in 5 years and 20-25% in 10 years^1^ ^,^ ^7^ ^,^ ^14^ ^,^ ^18^. Several strategies have been used to expand the possibility of resection and to ensure adequate liver remnants, such as parenchyma-preserving techniques, portal vein embolization, two-stage liver resection (LR), and ALLPS (Associating Liver Partition and Portal Vein Ligation for Staged Hepatectomy). Even with the use of these strategies, most patients with CRLM remain functionally or anatomically unresectable^20^.

The justification for performing liver transplantation (LT) in patients with CRLM regards the increase in the number of resectable patients by performing a total hepatectomy. However, LT in patients with CRLM was considered an absolute contraindication before 1995, due to unacceptable results obtained at the time. The first experience was reported by the European Liver Transplant Registry (ELTR), presenting survival rate of 62% in 1 year and 18% in 5 years^5^. It is worth mentioning that both the perioperative results of LT and the chemotherapy drugs available for the treatment of CRC in the late 1980s and early 1990s justify these aforementioned negative results. The poor results associated with organ scarcity resulted in the discontinuation of LT for CRLM^8^ ^,^ ^12^. Based on the data currently available, the International Liver Transplant Society (ILTS) is recommended to perform LT with a specific protocol for CLRM^11^.

Therefore, the aim of this study was to present a protocol proposal to guide the clinical use of LT in CRLM. This protocol needs to be validated in future studies.

## METHODS

This protocol was performed by two high-volume centers of LT and LR in Brazil: University Hospital of the Medical School of the University of São Paulo (HCFMUSP) and Hospital Adventista Silvestre/Hospital São Lucas. The elaboration of the protocol was conducted in four stages.

In the first stage, a search in the literature was performed in order to obtain the main published studies regarding LT for CRLM to date. In the second stage, an outline of the protocol was designed by the first two authors and the last author, based on the SECA trials from the University of Oslo^4^ ^,^ ^9^. In the third stage, 10 experts elaborated the last version of the protocol, adapted to the Brazilian reality. The fourth stage consisted of the protocol submitted for approval in the National Transplant System (SNT-Sistema Nacional de Transplantes) of the Brazilian Ministry of Health.

Brazilian centers were selected for inclusion in the multicentric research project, and a total of 30 patients underwent transplantation according to the criteria of this protocol and were referred to these centers by the SNT. Preoperative, intraoperative, and postoperative data were prospectively recorded on the REDCap platform^10^. The following pretransplantation data were analyzed: age, gender, body mass index (BMI), clinical performance, comorbidities, laboratory examinations, staging examinations, size and number of tumors, previous chemotherapy, response to chemotherapy, anatomopathological analysis of the primary tumor, time between diagnosis of CRC and LT, and type of LT (deceased donor or living donor). The number of patients referred for the LT evaluation, as well as the number of patients who effectively met the criteria and were included for undergoing LT and those who were excluded before the LT (due to not meeting the criteria) were also assessed. After the LT, disease-free survival and overall survival rate in 1, 3, and 5 years, immunosuppression protocol, rejection episodes, and need for retransplantation were analyzed.

## RESULTS

**Figure 1 -:**
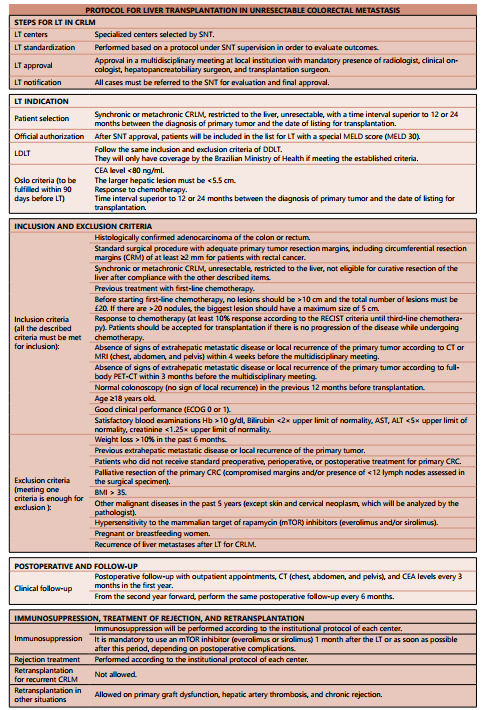
Protocol created by the authors for liver transplantation in unresectable colorectal metastasis.

**Figure 2 -:**
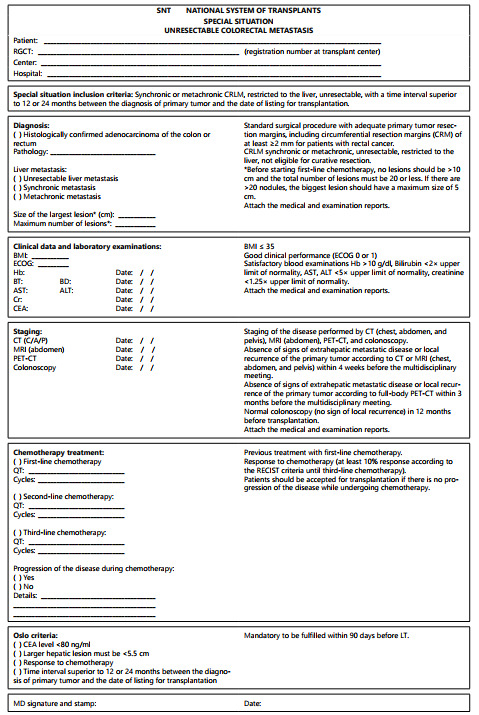
Document of SNT to be filled in to request a special situation for unresectable CRLM.

Figure 1 shows the LT protocol for CRLM proposed in this study by the authors. Figure 2 shows the document of SNT to be filled in to request a special situation for CRLM.

LT=liver transplantation; DDLT=deceased-donor liver transplantation; LDLT=living-donor liver transplantation; CRLM=colorectal liver metastasis; CRC=colorectal cancer; SNT=national system of transplants; CT= computed tomography; MRI=magnetic resonance imaging; PET-CT=positron emission tomography; ECOG=performance status of Eastern Cooperative Oncology Group.

LT=liver transplantation; DDLT=deceased-donor liver transplantation; LDLT=living-donor liver transplantation; CRLM=colorectal liver metastasis; CRC=colorectal cancer; SNT=national system of transplants; CT= computed tomography; MRI=magnetic resonance imaging; PET-CT=positron emission tomography; ECOG=performance status of Eastern Cooperative Oncology Group.

## DISCUSSION

In the past two decades, there has been improvement in the survival rates after LT by 20-30% and improvement in the imaging examinations; there was also the introduction of immunosuppressants with antineoplastic action (mTOR inhibitors)^15^ ^,^ ^17^. This technical progress combined with the peculiar transplantation scenario in Norway, which has more organ donors than recipients on the list, provided the ideal scenario for performing LT in CRLM. In the SECA I study, conducted from 2006 to 2011 at the University of Oslo, 21 patients underwent LT for CRLM, whose main inclusion criteria at the time consisted of good clinical performance (ECOG 0 or 1), complete resection of the primary tumor, and a minimum of 6 weeks of chemotherapy. The authors obtained an overall survival rate of 60% in 5 years and identified four clinical variables associated with a worse prognosis (Oslo criteria): tumor diameter >5.5 cm, CEA > 80 ng/ml, interval between resection and LT <2 years, and progression of the disease during chemotherapy^9^.

The same group from Oslo continued the investigation of LT for CRLM through the SECA II study. From 2012 to 2016, 15 patients were transplanted with restrictive criteria in order to obtain a result similar to other causes of LT. Several criteria were included for the performance of LT, such as the Oslo criteria, the nonresectability due to partial hepatectomy, and the radiological tumor response after chemotherapy. The authors obtained an overall survival rate of 100% in 1 year, 83% in 3 years, and 83% in 5 years. Disease-free survival rate obtained was 53% in 1 year, 44% in 3 years, and 35% in 3 years. The main site of recurrence was pulmonary and most of them were fit for resection; therefore, the high rates of recurrence had less influence on the survival rate of patients^4^.

Both the aforementioned Norwegian studies have major importance in the “Transplant Oncology,” a term used to describe LT as a treatment option for hepatobiliopancreatic neoplasms. Recently, multiple centers in Europe and in the United States have started to perform LT for CRLM^11^ ^,^ ^19^. Fernandes et al. were pioneers in performing the first LT with a living donor in Latin America in a patient with CRLM, in agreement with the Oslo criteria^6^.

The exclusion of transplantation in cases of right colon tumor and/or the presence of positive BRAF is a topic to be discussed. Mutation-positive BRAF is considered a risk factor and is associated with worse outcomes after transplantation. Tumors of the right colon also have a worse prognosis, precisely due to their higher frequency of positive BRAF^16^. Clinical studies that are still in progress present heterogeneity regarding these items and, therefore, we chose to maintain them in our protocol until further studies. In Norway (NCT01479608, NCT02215889, and NCT03494946) and Germany (NCT03488953), the studies do not adopt these exclusion criteria, while in France (NCT02597348), Canada (NCT02864485), and Italy (NCT03803436), positive BRAF is the exclusion criteria^3^ ^,^ ^11^.

The regulation of this protocol is in progress in the SNT for validation in the Brazilian national territory^2^.

## CONCLUSION

An LT protocol for colorectal unresectable metastasis was created to standardize the treatment and to enable a better evaluation of not only surgical results but also disease-free survival and overall survival of patients with CRLM.
